# Transcription factor ONECUT3 regulates HDAC6/HIF-1α activity to promote the Warburg effect and tumor growth in colorectal cancer

**DOI:** 10.1038/s41419-025-07457-8

**Published:** 2025-03-03

**Authors:** Ruixue Huo, Weihan Li, Hao Wu, Kexin He, Hao Wang, Shan Zhang, Shu-Heng Jiang, Rongkun Li, Junli Xue

**Affiliations:** 1 Department of Oncology, Shanghai East Hospital, School of Medicine, Tongji University, Shanghai, P.R. China; 2 State Key Laboratory of Systems Medicine for Cancer, Shanghai Cancer Institute, Ren Ji Hospital, School of Medicine, Shanghai Jiao Tong University, Shanghai, P.R. China; 3 Chest Oncology Department, Cancer Institute of Jiangsu University, Affiliated Hospital of Jiangsu University, Zhenjiang, P.R. China

**Keywords:** Colon cancer, Cancer metabolism

## Abstract

The Warburg effect, also known as aerobic glycolysis, plays a crucial role in the onset and progression of colorectal cancer (CRC), although its mechanism remains unclear. In this study, bioinformatics analysis of public databases combined with validation using clinical specimens identified the transcription factor ONECUT3 as a key regulator related to the Warburg effect in CRC. Functionally, silencing ONECUT3 reverses the Warburg effect and suppresses tumor growth. Importantly, ONECUT3 promotes tumor growth in a glycolysis-dependent manner through hypoxia-inducible factor 1α (HIF-1α). Mechanistically, ONECUT3 does not directly regulate the expression of HIF-1α but instead inhibits its acetylation via histone deacetylase 6 (HDAC6). This deacetylation enhances the transcriptional activity of HIF-1α, ultimately upregulating multiple glycolysis-related genes downstream of HIF-1α, thereby driving the Warburg effect and facilitating tumor growth in CRC. These findings reveal a novel mechanism by which ONECUT3 regulates the Warburg effect in CRC and suggest that targeting ONECUT3 may offer a promising therapeutic strategy for CRC.

## Introduction

Colorectal cancer (CRC) is a highly prevalent malignancy, ranking third in terms of incidence and second in cancer-related mortality worldwide. Despite advancements in cancer diagnosis and treatment, the incidence and mortality rates of CRC continue to rise, particularly in developing countries and among young adults (under 50 years old). It is projected that by 2040, there will be 3.2 million new cases and 1.6 million deaths from CRC [1], which poses a significant threat to human survival and imposes a substantial economic burden. Therefore, a deeper understanding of the specific molecular mechanisms involved in CRC and developing targeted drugs could potentially improve the disease prognosis.

Unlike normal tissues, tumor cells undergo metabolic reprogramming, a hallmark characteristic of cancer [2, 3]. Despite sufficient oxygen availability, tumor cells predominantly metabolize glucose through glycolysis, resulting in lactate accumulation. This phenomenon, referred to aerobic glycolysis, is commonly known as the Warburg effect [4]. Numerous studies have demonstrated that the Warburg effect remodels the tumor microenvironment and plays a crucial role in the initiation, progression, metastasis, and prognosis of CRC [5–9]. Research has confirmed that transcription factors can regulate tumor glycolysis through various mechanisms, including modulation of the glycolysis phase, expression of related genes, and regulation of key glycolytic enzyme activities [10–14]. The glycolytic process is primarily regulated by hypoxia-inducible factor 1α (HIF-1α). Under hypoxic conditions, HIF-1α forms a heterodimer with HIF-1β, which then translocates to the nucleus and transcriptionally activates the transcription of downstream target genes (e.g., *SLC2A1* (*GLUT1*), etc.). The regulation of HIF-1α in CRC is influenced by frequently mutated genes, including *APC*, *RAS*, and *TP53*, as well as by epigenetic modifications [5, 15–19]. However, the specific mechanism underlying the regulation of HIF-1α in CRC is still unclear.

The ONECUT family, comprising ONECUT1 (HNF6), ONECUT2, and ONECUT3, contains a single “CUT” DNA-binding domain and an atypical homologous domain, and functions as translational factors [20]. The three members of the ONECUT family, which are highly conserved from Drosophila to humans, play a crucial role in regulating the development of various tissues derived from the ectoderm or endoderm, as well as in the regulation of numerous gene families [21, 22]. Previous studies have reported that ONECUT1 promotes colon cancer cell proliferation and liver metastasis by enhancing cell adhesion ability and inhibiting apoptosis [23]. In gastric cancer, upregulation of ONECUT2 expression promotes cell migration, invasion, epithelial-mesenchymal transition, and tumor growth [24]. Similarly, elevated ONECUT2 expression is associated with lymph node metastasis [25] and poorer prognosis in CRC [26]. However, as a relatively new member of the ONECUT family [27], the role of ONECUT3 in cancer, particularly regarding glucose metabolism reprogramming, has been less extensively studied.

In this study, we demonstrated that ONECUT3 mediates HIF-1α deacetylation through histone deacetylase 6 (HDAC6), leading to the activation of HIF-1α transcription and its downstream glycolysis-related genes, thereby enhancing the Warburg effect and promoting tumor growth in CRC. The ONECUT3-HDAC6-HIF-1α axis represents a potential therapeutic target for modulating aerobic glycolysis in CRC.

## Results

### ONECUT3 is a key transcription factor related to glycolysis in CRC

**Fig. 1:**
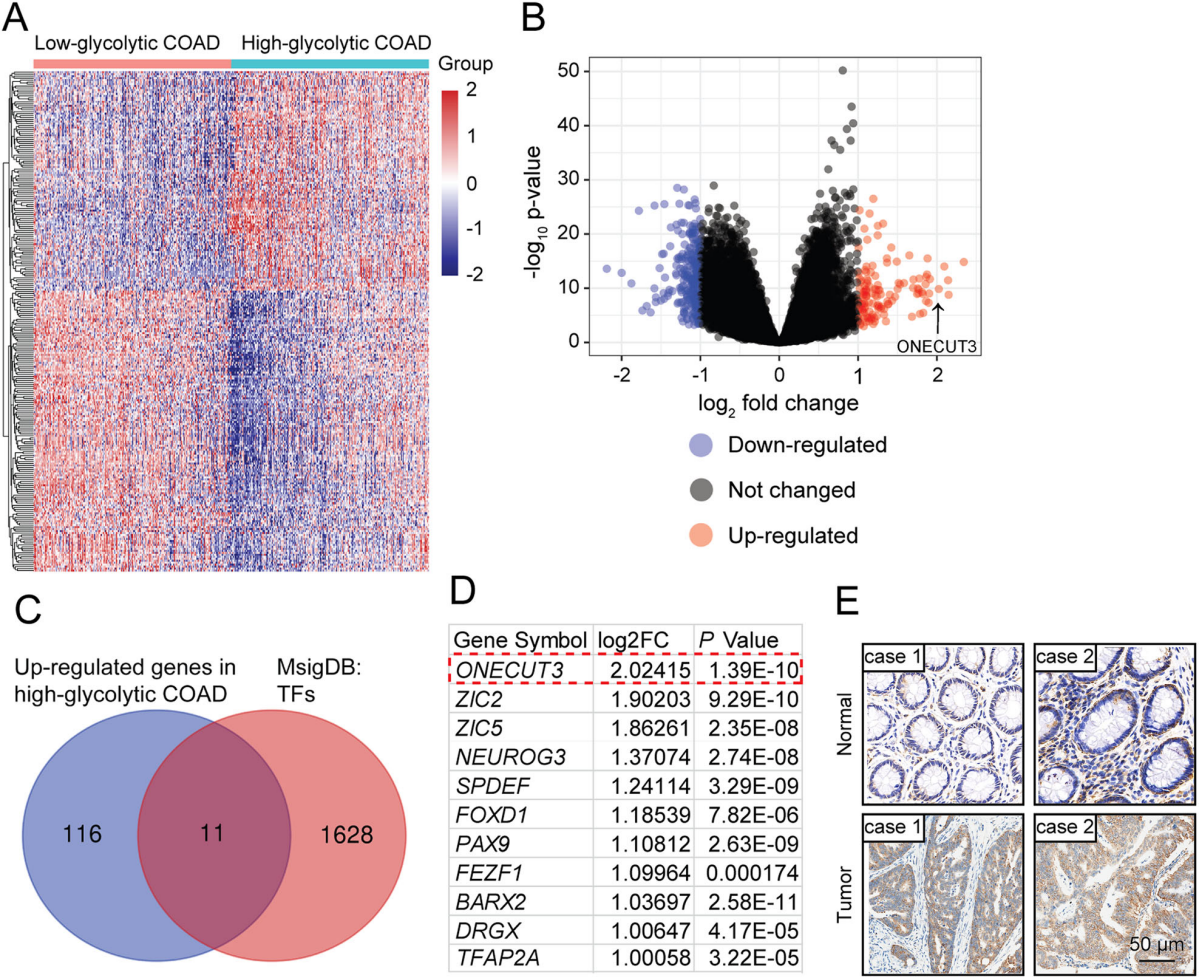
ONECUT3 is a key transcription factor related to glycolysis in CRC. **A** Based on the known glycolysis-related gene clusters, TCGA-COAD samples were hierarchically clustered into high-glycolysis and low-glycolysis groups. **B** Screening for differentially expressed genes in the two groups. **C** The 127 upregulated genes were intersected with MsigDB: TF to obtain 11 transcription factors. **D** Ranking of log2|FC| values of 11 transcription factors. **E** Representative IHC images showed the level of ONECUT3 expression in CRC tissues and paracancerous tissues (scale bar: 50 μm).

To elucidate the genes associated with aerobic glycolysis in colon cancer, RNA-sequencing (RNA-seq) data from the Colon Adenocarcinoma (COAD) subset of The Cancer Genome Atlas (TCGA) database were analyzed. In the TCGA-COAD database, colon cancer samples were categorized into two groups based on the expression levels of glycolysis-related genes: the high-glycolysis group and the low-glycolysis group (Fig. 1A). A total of 400 differentially expressed genes (DEGs) were identified using a significance threshold of Log_2_|Fold change | ≥1 and *p* < 0.05. Among these, 127 genes were up-regulated in the high-glycolysis group, including 11 genes that encode transcription factors (Fig. 1B, C). Among the 11 genes, *ONECUT3* (Fig. 1D) was identified as the most significant DEG. Therefore, *ONECUT3* was selected as the key gene to investigate the glycolytic pathway in colon cancer. Subsequently, immunohistochemical staining was performed to determine the expression of ONECUT3 in both colon cancer tissues and adjacent normal tissues. The results revealed a significant increase in ONECUT3 expression in CRC tissues (Fig. 1E and Supplementary Fig. 1), suggesting a potential regulatory role of ONECUT3 in CRC through glycolysis.

### Regulation of glycolytic metabolism in CRC cells by ONECUT3

**Fig. 2:**
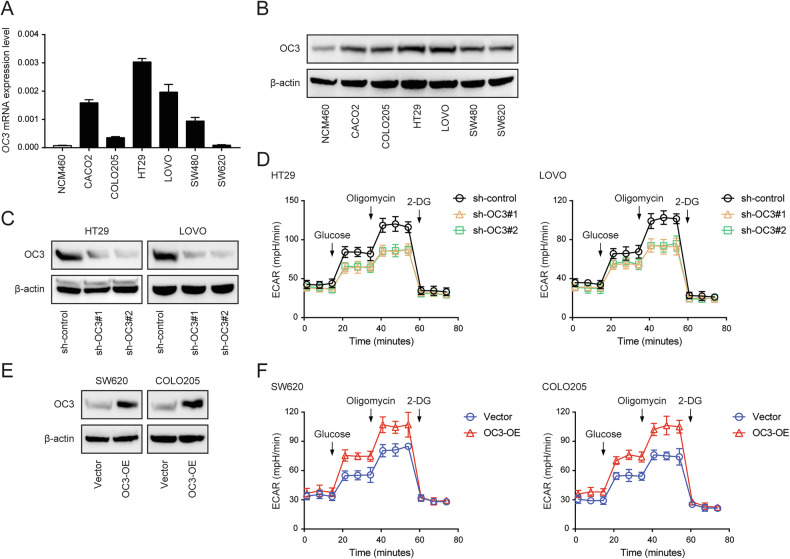
Regulation of glycolytic metabolism in CRC cells by ONECUT3. **A**, **B** qRT-PCR (**A**) and western blot (**B**) to detect ONECUT3 expression in colon cancer cell lines (CACO2, COLO205, HT29, LOVO, SW480, SW620) and normal colon epithelial line NCM460. **C** *ONECUT3* was knocked down in HT29 and LOVO and the efficiency was verified. **D** The results of the Seahorse XF Bioanalyzer assay showed the ECAR results after knocking down *ONECUT3* in colon cancer cells. **E** *ONECUT3* was overexpressed in SW620 and COLO205, and the efficiency was verified. **F** The results of Seahorse XF Bioanalyzer showed the ECAR results after overexpression of *ONECUT3* in colon cancer cells. (OC3 ONECUT3, ECAR extracellular acidification rate).

Initially, we detected the mRNA and protein expression of ONECUT3 in six colon cancer cell lines (CACO2, COLO205, HT29, LOVO, SW480, SW620) and a regular colon epithelial cell line (NCM460) (Fig. 2A, B). The expression of *ONECUT3* was highest in HT29 and LOVO cell lines while lowest in SW620 and COLO205 cell lines. Subsequently, *ONECUT3* was knocked down in the high-expressing cell lines (HT29 and LOVO) and overexpressed in the low-expressing cell lines (SW620 and COLO205), as depicted in Fig. 2C, E. We conducted an extracellular acidification rate (ECAR) assay in colon cancer cells to examine the regulatory role of ONECUT3 in glycolysis. The results demonstrated a significant decrease in the ECAR, an indicator of glycolysis, following *ONECUT3* knockdown (Fig. 2D) and a substantial increase after *ONECUT3* overexpression in colon cancer cells (Fig. 2F). These findings indicate that ONECUT3 promotes the glycolytic metabolism of CRC cells.

### ONECUT3 promotes the growth of CRC cells in a glycolysis-dependent manner

**Fig. 3:**
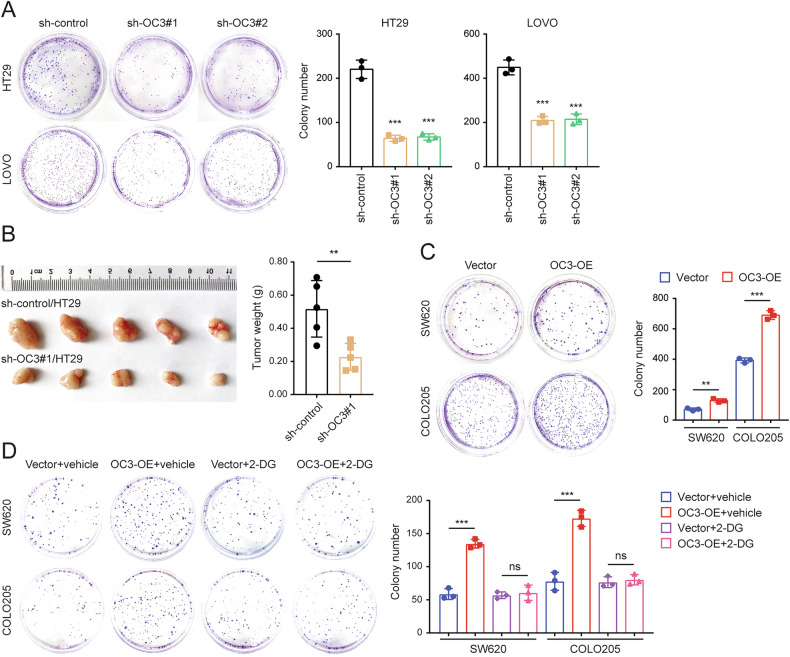
ONECUT3 promotes the growth of CRC cells inc a glycolysis-dependent manner. **A** colony-forming assay showed the proliferation of HT29 and LOVO cells after knocking down *ONECUT3*. **B** Subcutaneous xenograft model showed tumor growth after knocking down *ONECUT3* (*n* = 5). **C** colony-forming assay showed the proliferation of SW620 and COLO205 cells after overexpression of *ONECUT3*. **D** colony-forming assay showed the proliferation of *ONECUT3-*overexpressed cells after using the glycolysis inhibitor 2-DG. (OC3 ONECUT3; **p < 0.01, ***p < 0.001, ns no significance).

We investigated whether ONECUT3 could influence the proliferation of CRC cells through the Warburg effect, which is known to enhance tumor cell proliferation [5, 6, 8, 13, 14, 18, 28]. In the colony-forming assay, knockdown of *ONECUT3* significantly inhibited the proliferation of HT29 and LOVO cell (Fig. 3A), while overexpression of *ONECUT3* significantly promoted the proliferation of SW620 and COLO205 cell lines (Fig. 3C). The promotion of proliferation by *ONECUT3* overexpression was largely abolished by the glycolysis inhibitor 2-Deoxy-D-Glucose (2-DG) (Fig. 3D). We utilized a nude mouse subcutaneous transplantation tumor model to verify the effect of ONECUT3 on tumor growth in vivo. Likewise, the knockdown of ONECUT3 significantly reduced the weight of the tumors, indicating a significant inhibition of CRC growth by ONECUT3 (Fig. 3B). These results indicated that ONECUT3 promotes tumor growth in a glycolysis-dependent manner.

### ONECUT3 enhances the transcriptional activity of HIF-1α and regulates the expression of its downstream glycolytic enzymes

**Fig. 4:**
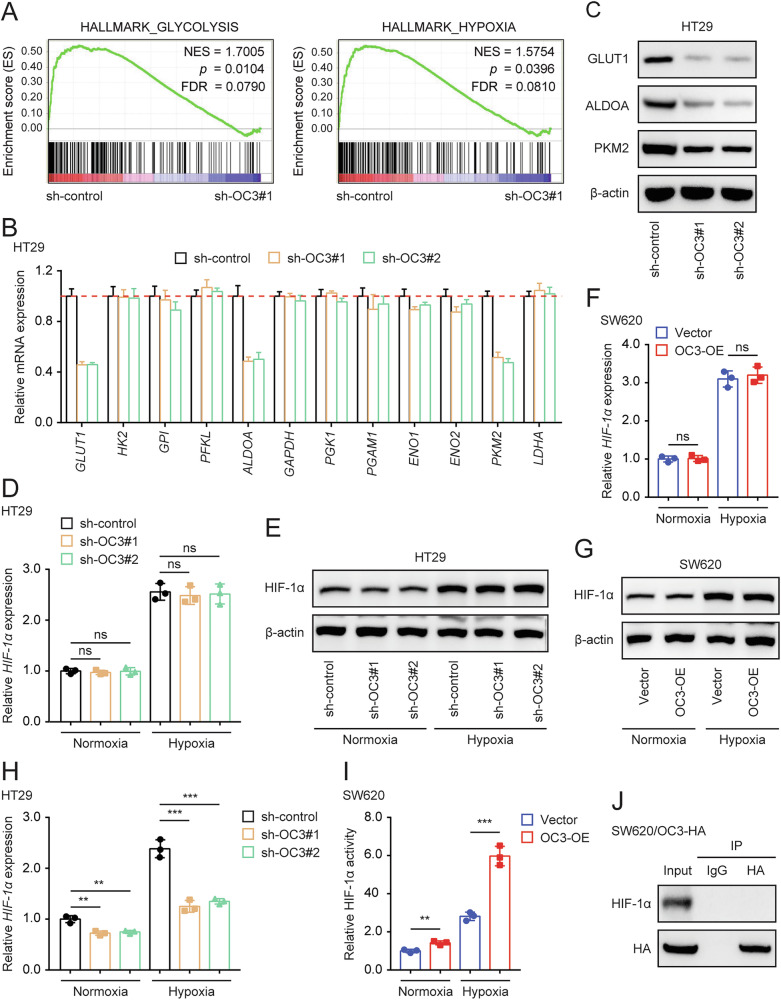
ONECUT3 enhances the transcriptional activity of HIF-1α and regulates the expression of its downstream glycolytic enzymes. **A** After the knockdown of *ONECUT3* in HT29 cells, GSEA enrichment of transcriptome sequencing. **B**, **C** qRT-PCR (**B**) and western blot (**C**) showed mRNA and protein expression of the downstream glycolysis-related enzymes of HIF-1α after knockdown of *ONECUT3*. **D–G** qRT-PCR (**D**) and western blot (**E**) showed the HIF-1α expression after the knockdown of *ONECUT3*; qRT-PCR (**F**) and western blot (**G**) showed the HIF-1α expression after the overexpression of *ONECUT3*. **H**, **I** HIF-1 alpha Transcription Factor Assay kit showed the HIF-1α transcriptional activity after knockdown of *ONECUT3* (**H**) and overexpression of *ONECUT3* (**I**) in colon cancer cells. **J** Co-IP assay showed that there was no direct interaction between ONECUT3 and HIF-1α. OC3 ONECUT3; **p < 0.01, ***p < 0.001, ns no significance.

To determine if transcription factor ONECUT3 can directly regulate the transcription of glycolytic genes, we utilized online databases including JASPAR, ALGGEN, and hTFtarget to predict the genes that ONECUT3 regulates. However, our search did not identify any glycolysis-related genes that are directly targeted by ONECUT3. Subsequently, RNA sequencing was employed to analyze the changes in the transcriptome resulting from ONECUT3 knockdown in colon cell line HT29 (sh-control vs sh-*OC3*#1). Gene Set Enrichment Analysis (GSEA) results indicated the close involvement of ONECUT3 in glycolytic and hypoxic signaling pathways (Fig. 4A). Subsequently, the expression of key genes in the glycolytic signaling pathway was assessed at both the RNA and protein levels. RT-qPCR and Western Blot experiments confirmed that GLUT1 (*SLC2A1*), *ALDOA*, and *PKM2* expression were reduced after *ONECUT3* knockdown (Fig. 4B, C). These enzymes are downstream targets of HIF-1α and play a crucial role in the glycolytic pathway[29–33]. Given that HIF-1α is a key regulator of glycolytic signaling, it is hypothesized that ONECUT3 may be involved in regulating glycolytic metabolism through HIF-1α.

We measured the expression level of HIF-1α after knocking down or overexpressing ONECUT3. However, the subsequent experimental results showed that knockdown or overexpressing ONECUT3 in colon cancer cells did not affect the mRNA or protein expression of HIF-1α (Fig. 4D–G). Therefore, we investigated whether ONECUT3 affects the expression of downstream genes of HIF-1α by regulating its transcriptional activity. Experiments using the HIF-1 alpha Transcription Factor Assay kit revealed that the knockdown of ONECUT3 significantly suppressed HIF-1α transcriptional activity (Fig. 4H) while overexpressing ONECUT3 enhanced HIF-1α transcriptional activity (Fig. 4I). Since both ONECUT3 and HIF-1α are transcription factors, we hypothesized that they can directly bind and interact with each other in the nucleus. However, the Co-immunoprecipitation (Co-IP) assay did not provide evidence of direct interaction between ONECUT3 and HIF-1α (Fig. 4J). This suggests that ONECUT3 could enhance the transcriptional activity of HIF-1α and influence the expression of multiple downstream glycolytic enzymes, but not through direct interaction with HIF-1α.

### ONECUT3 regulates HIF-1α transcriptional activity through HDAC6

**Fig. 5:**
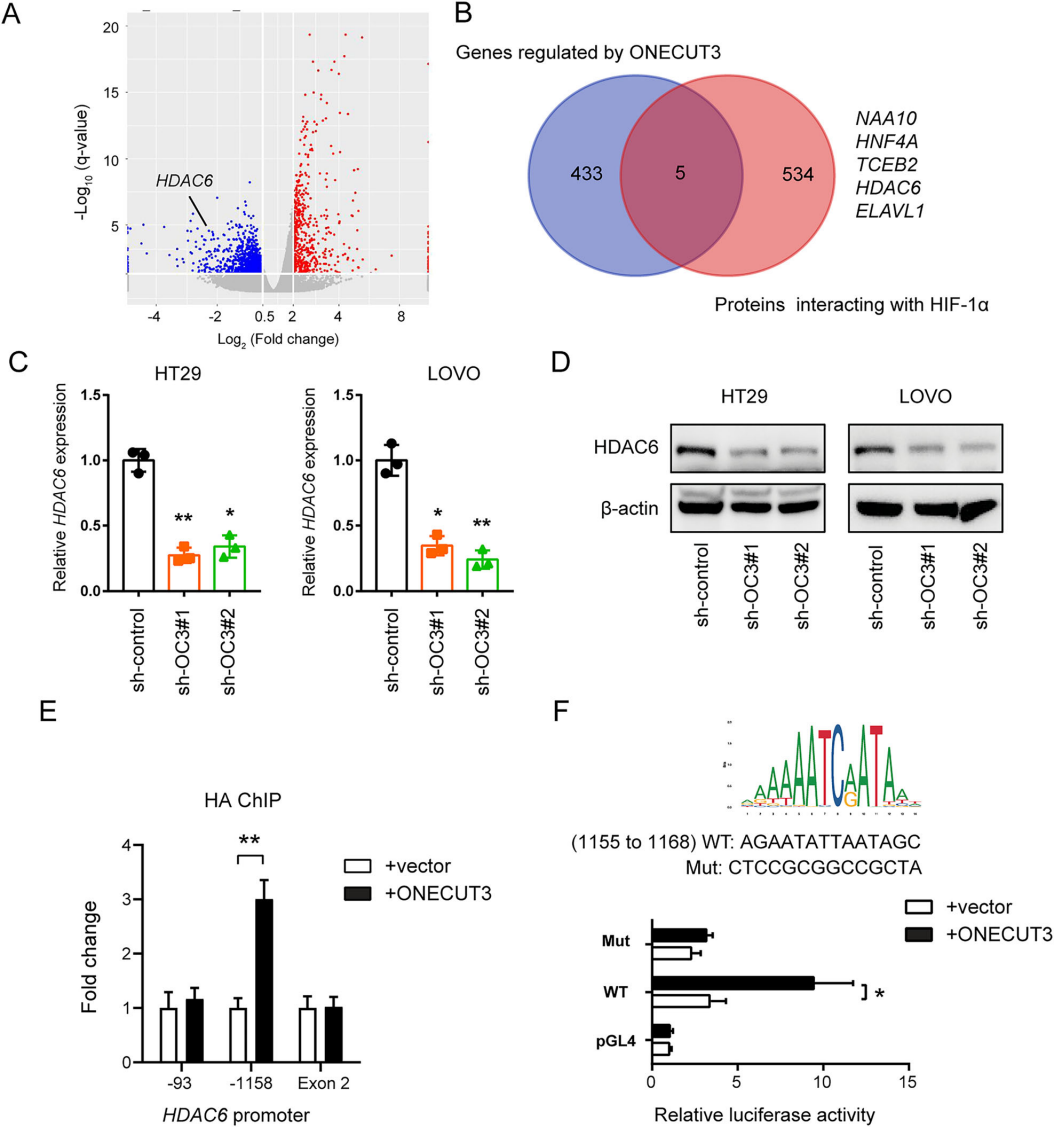
ONECUT3 regulates HIF-1α transcriptional activity through HDAC6. **A** Volcano plot showed differentially expressed genes (DEGs) caused by *ONECUT3* knockdown. **B** Five genes (*NAA10*, *HNF4A*, *TCEB2*, *HDAC6* and *ELAVL1*) were identified after intersection of proteins interacting with HIF-1α and *ONECUT3*-regulated genes (n = 438). **C** qRT-PCR results showed the expression of *HDAC6* after knockdown of *ONECUT3* in HT29 and LOVO cells. **D** Western Blot results showed protein expression of HDAC6 after *ONECUT3* knockdown. **E** ChIP-PCR analysis revealed that ONECUT3 was specifically bound to the HDAC6 promoter region at −1158, while no significant binding was observed at the -93 region or Exon 2, the latter serving as a negative control. **F** Luciferase reporter gene assay demonstrated that ONECUT3 transcriptionally regulated the *HDAC6* gene. *p < 0.05, **p < 0.01, ns: no significance.

To elucidate the interaction mechanism between ONECUT3 and HIF-1α, we initially screened 438 genes regulated by ONECUT3 using RNA-seq analysis (Fig. 5A). Subsequently, we identified 539 proteins interacting with HIF-1α through Co-IP and mass spectrometry. By performing a Venn analysis (Fig. 5B), we obtained five genes (*NAA10*, *HNF4A*, *TCEB2*, *HDAC6*, and *ELAVL1*) in both groups. Previous studies have shown that inhibiting HDAC6 selectively can decrease HIF-1-mediated transcription in hypoxic conditions. Additionally, HDAC inhibitors can suppress tumor angiogenesis and the expression of the HIF-1α protein [34, 35]. Based on these findings, we hypothesized that ONECUT3 may regulate the transcriptional activity of HIF-1α by modulating HDAC6. In supporting our hypothesis, the knockdown of *ONECUT3* significantly reduced HDAC6 expression in both qRT-PCR and Western Blot analyses (Fig. 5C, D). Further investigations using Chromatin Immunoprecipitation-Polymerase Chain Reaction (ChIP-PCR) demonstrated that ONECUT3 binds to the promoter region of *HDAC6* at the -1158 site. To further confirm this finding, we mutated this promoter region of *HDAC6* and conducted luciferase reporter gene assays. The results indicated an increased DNA-binding activity in the wild-type HDAC6 promoter, whereas the reporter activity decreased following the mutation of the binding site. In conclusion, our findings suggest that ONECUT3 interacts with the *HDAC6* promoter region and directly regulates its transcription in CRC cells (Fig. 5E, F).

### ONECUT3 facilitates the deacetylation of HIF-1α and enhances its transcriptional activity through HDAC6

**Fig. 6:**
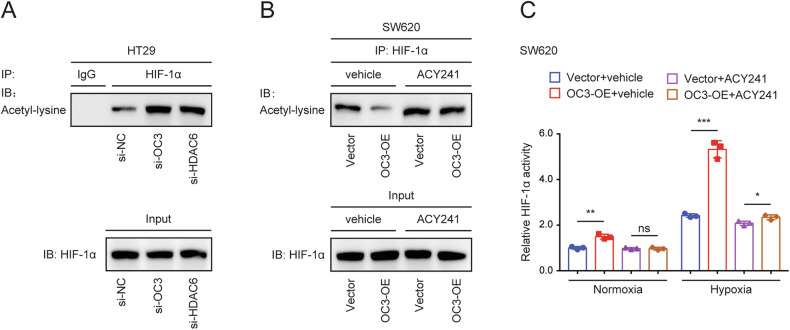
ONECUT3 facilitates the deacetylation of HIF-1α and enhances its transcriptional activity through HDAC6. **A** Western Blot showed HIF-1α acetylation level by detecting pan-acetylation antibody after knockdown of *ONECUT3* or *HDAC6* in HT29 cells. **B** Western blot analysis revealed the acetylation level of HIF-1α following the overexpression of *ONECUT3* or the HDAC6 inhibitor (ACY241) in SW620 cells. **C** HIF-1 alpha Transcription Factor Assay kit showed the transcriptional activity of HIF-1α after overexpression of *ONECUT3* or/and inhibition of *HDAC6* in SW620 cells. *p < 0.05, **p < 0.01, ***p < 0.001. ns: no significance.

To validate the involvement of HDAC6 in regulating HIF-1α transcriptional activity by ONECUT3, we performed knockdown experiments targeting either *ONECUT3* or *HDAC6* in CRC cells. Subsequently, HIF-1α was enriched through Co-IP. Post-translational modifications are known to affect the activity of HIF-1α, with acetylation leading to decreased stability[36]. In normoxic conditions, the hydroxylation of two proline residues and the acetylation of a lysine residue on the oxygen-dependent degradation domain of HIF-1α facilitates its binding to the pVHL E3 ligase complex, resulting in its degradation through the ubiquitin-proteasome pathway[37]. The acetylation level of HIF-1α was determined using a panacetylation antibody. Consistent expression of HIF-1α resulted in a significant increase in its acetylation level following the knockdown of *ONECUT3* or *HDAC6* (Fig. 6A). Conversely, overexpression of *ONECUT3* led to a substantial reduction in the acetylation level of HIF-1α (Fig. 6B). Additionally, the facilitative effect of *ONECUT3* overexpression on HIF-1α deacetylation could be reversed by ACY241, an inhibitor of HDAC6 (Fig. 6B). Notably, inhibition of HDAC6 significantly eliminated the enhanced HIF-1α transcriptional activity in *ONECUT3* overexpressing cells (Fig. 6C). These results indicate that the regulatory impact of ONECUT3 on HIF-1α deacetylation and transcriptional activity is mediated by HDAC6.

### Expression of ONECUT3 in CRC and its clinical significance

**Fig. 7:**
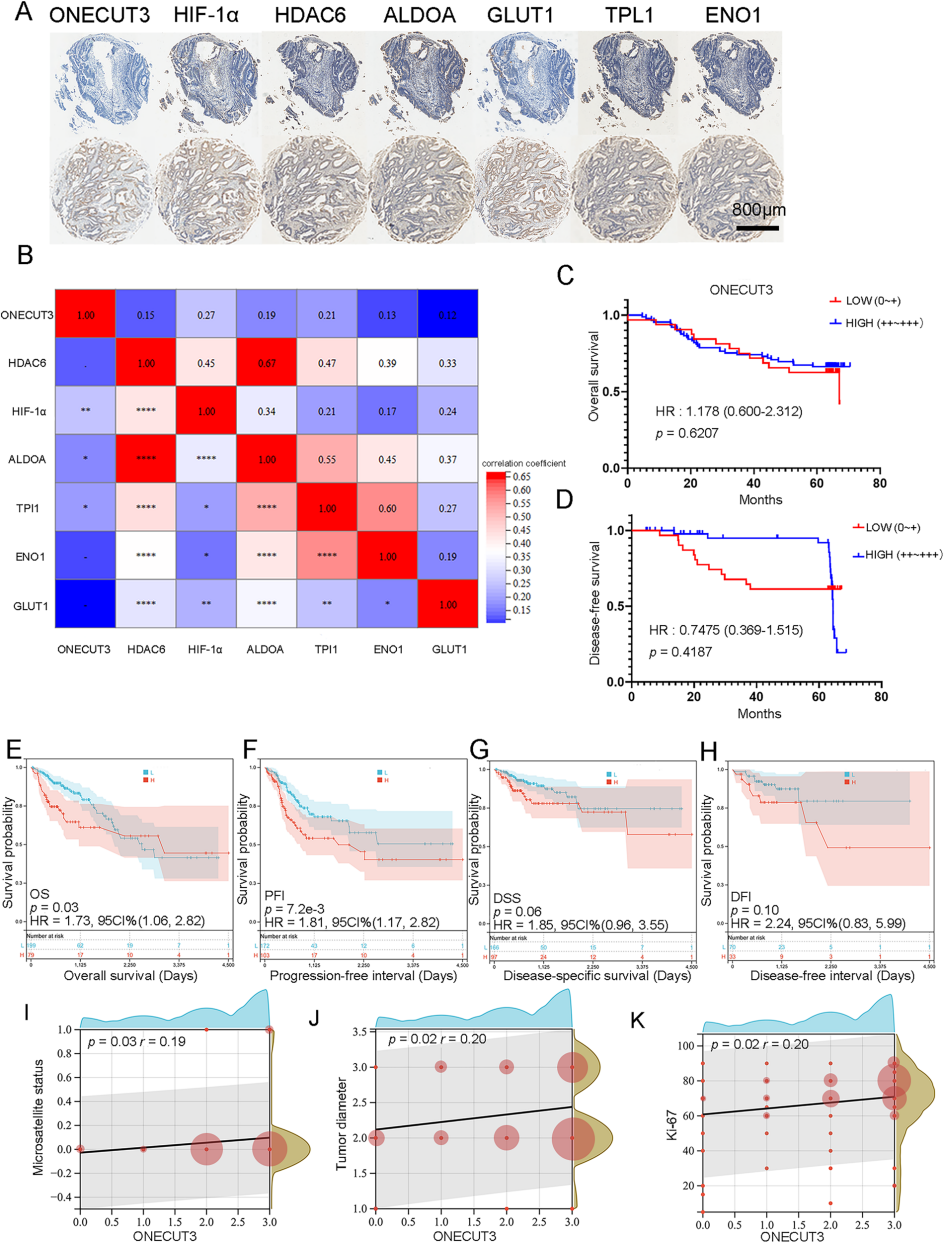
Expression of ONECUT3 in CRC and its clinical significance. **A** Representative IHC image of CRC tissue microarrays. The upper column showed relatively negative staining, and the lower one showed relatively positive staining. **B** Correlation analysis of ONECUT3 with the expression of HIF-1a, HDAC6, and glycolysis-related proteins (ALDOA, TPI1, GLUT1, and ENO1) in tissue microarrays. **C**, **D** OS and DFS analysis of ONECUT3 in Renji cohort. **E**–**H** OS analysis (**E**), PFI analysis (**F**), DSS analysis (**G**), and DFI analysis (**H**) of ONECUT3 in TCGA- COAD database. **I**, **K** Expression of ONECUT3 was significantly correlated with microsatellite status (**I**), tumor diameter (**J**), and Ki-67 level (**K**). OS Overall survival, DFS disease-free survival, PFI progression-free survival, DSS Disease-specific survival, DFI disease-free interval. *p < 0.05, ** *p* < 0.01, *** *p* < 0.001.

To confirm the clinical significance of ONECUT3, we conducted immunohistochemical staining on microarrays of CRC patients from the Renji Hospital cohort (Fig. 7A) to examine the expression of ONECUT3, HIF-1A, HDAC6, and glycolysis-related proteins. The expression of ONECUT3 showed a positive correlation with specific glycolysis-related proteins (ALDOA, TPI1) and HIF-α (*p* < 0.05). Additionally, both HIF-α and HDAC6 exhibited a positive correlation with the expression of glycolysis-related proteins (ALDOA, TPI1, GLUT1, and ENO1) (*p* < 0.05). The expression of HIF-α and HDAC6 showed a positive correlation (*p* < 0.05) (Fig. 7B). We conducted a correlation analysis between ONECUT3 and clinicopathologic parameters. The results showed a significant correlation between the expression of ONECUT3 and microsatellite status, tumor diameter, and Ki-67 level. However, there was no correlation with the TNM stage, *KRAS* mutation, vascular invasion, perineural invasion, histological grading, and tumor site. Specifically, higher ONECUT3 expression was associated with microsatellite instability status, larger tumor diameters, and higher Ki-67 levels (Fig. 7I–K). However, in the survival analysis, there was no association between ONECUT3 expression and overall survival (OS) (*p* = 0.6207) or disease-free survival (DFS) (*p* = 0.4178) (Fig. 7C, D). We further analyzed the data from the TCGA-COAD. The results revealed a significant correlation between the ONECUT3 expression levels and patients’ overall survival (OS) and progression-free interval (PFI). Notably, higher ONECUT3 expression was linked to shorter durations of OS and PFI. While there is no significant association was found between ONECUT3 and disease-specific survival (DSS) or disease-free interval (DFI), the significant relationships with OS and PFI highlight the potential prognostic implications of ONECUT3 in colon cancer progression (Fig. 7E–H).

## Discussion

**Fig. 8:**
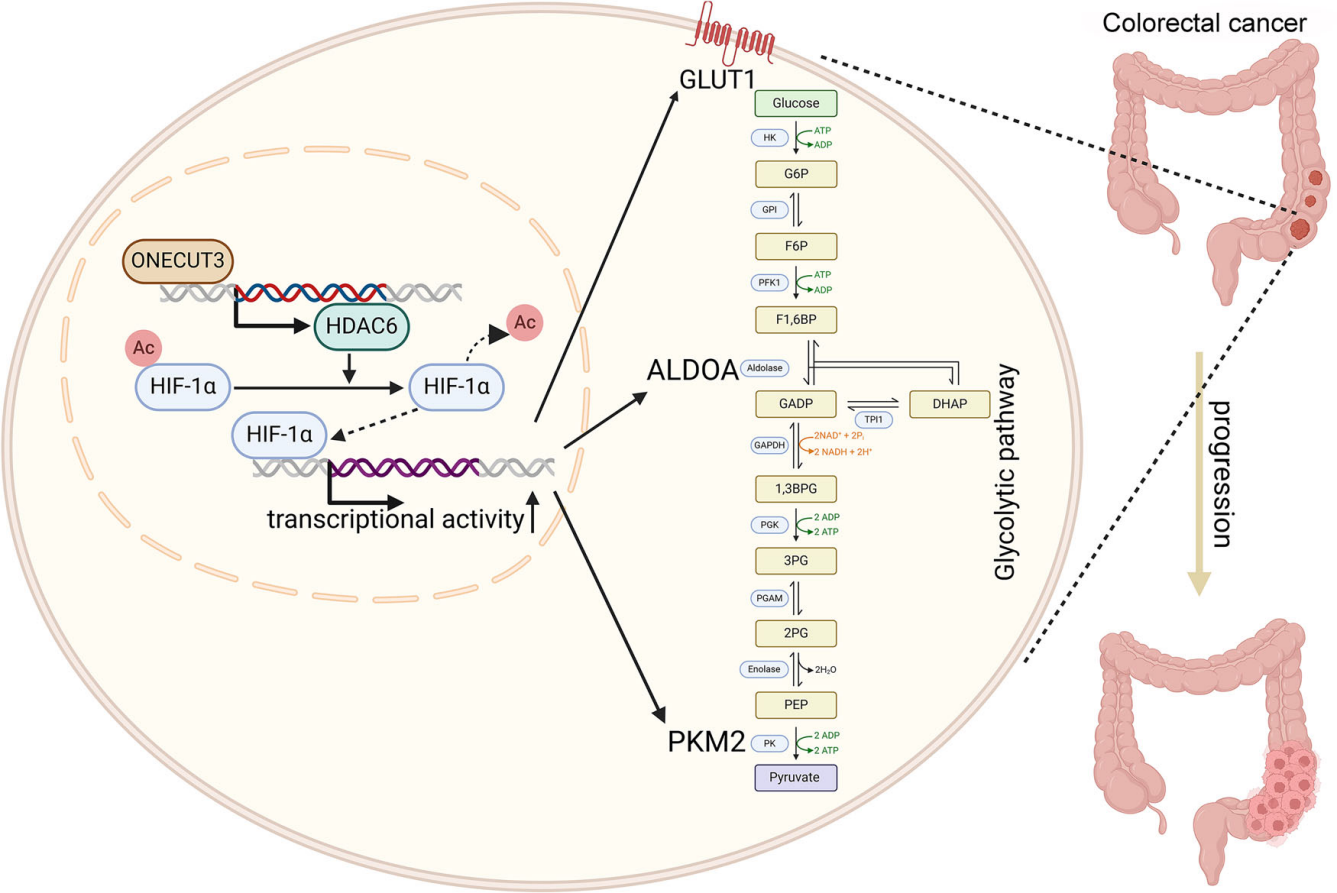
Schematic representation of ONECUT3-HDAC6-HIF-1α axis that regulates proliferation in CRC. ONECUT3 regulates HIF-1α deacetylation through HDAC6, enhancing HIF-1α transcriptional activity, which further promotes glycolysis in CRC cells and ultimately contributes to tumor progression. The original figure was created with BioRender.com.

The Warburg effect contributes to the progression of various malignant tumors. However, the exact mechanism by which it is regulated in CRC remains unclear. Our research, utilizing both in vivo and in vitro experiments, has revealed that ONECUT3 can influence the Warburg effect in CRC. We have also elucidated the specific mechanism by which ONECUT3 regulates the transcriptional activity of HIF1α via HDAC6 (Fig. 8). Given the limited advancements of targeted therapies for CRC, the identification of ONECUT3 may represent a promising target for CRC treatment.

The *ONECUT3* gene is located on human chromosome 19p13.3 and encodes the transcription factor ONECUT3. As a homolog of ONECUT1, ONECUT3 plays a vital role in early bile duct development in zebrafish, neuronal differentiation, endodermal differentiation of the human pancreas, and hearing development in Drosophila [20, 38–41]. Our research has provided new insights into the role of ONECUT3 in the aerobic glycolysis of CRC. We found that *ONECUT3* was significantly upregulated in the high-glycolytic group according to the TCGA database, and its expression was notably higher in colon cancer tissues compared to adjacent non-cancerous tissues. Further experiments showed that knocking down *ONECUT3* inhibited the proliferation and glycolysis of colon cancer cells, while overexpressing *ONECUT3* enhanced these processes. Additionally, the glycolysis inhibitor 2-DG mitigated the proliferative effects of *ONECUT3* overexpression in colon cancer cells. Our clinical study also revealed a positive correlation between ONECUT3 expression and microsatellite instability (MSI). Given that CRC patients with MSI tend to respond more favourably to immunotherapy, further exploration of the relationship between ONECUT3 and sensitivity to immunotherapy is necessary. In recent years, there have been few significant advancements in immunotherapy for CRC; enhancing its effectiveness remains a challenge [42]. Besides developing new immunotherapy targets, investigating innovative combination strategies could also improve treatment outcomes, as glycolytic metabolism influences both tumor cells and the immune components of the tumor microenvironment [43]. Similar to our findings, research in pancreatic cancer has shown that ONECUT3 affects tumor glycolysis and inhibits CD8 + T cell infiltration, as well as the efficacy of anti-PD-1 therapy [44]. Thus, ONECUT3, identified as a regulator of the Warburg effect in CRC in this study, maybe a promising candidate for combination with immunotherapy to improve its effectiveness and address existing challenges in treating CRC.

In our investigation of mechanisms, GSEA enrichment analysis indicated a strong association between *ONECUT3* and the hypoxia signaling pathway. Immunohistochemical results from CRC microarrays confirmed a positive correlation between ONECUT3 and HIF-1α and its downstream glycolysis-related proteins. HIF-1α is widely recognized as a critical effector molecule that enables cells to adapt to hypoxic stress and regulates tumor glycolytic metabolism [10]. It plays a significant role in controlling tumor cell glycolysis through the transcriptional regulation of various metabolic enzymes within the glycolytic pathway, including glucose transporters 1 and 3 (GLUT1/3), pyruvate dehydrogenase kinase 1 (PDK-1), lactate dehydrogenase A (LDH-A), and pyruvate kinase M2 (PKM-2). The upregulation of HIF-1α during tumor development can enhance the Warburg effect [45]. Given the importance of HIF-1α in aerobic glycolysis, we hypothesized that ONECUT3 might regulate tumor glycolytic metabolism through HIF-1α. However, cellular assays revealed that neither the knockdown nor overexpression of ONECUT3 affected the expression of HIF-1α at the mRNA or protein levels. Further investigations demonstrated that ONECUT3 regulates the transcriptional activity of HIF-1α, as evidenced by the downregulation of several HIF-1α target genes (*GLUT1*, *ALDOA*, *PKM2*) following the knockdown of ONECUT3. It is important to note that gene regulation is a complex process influenced by various transcription factors, and transcription patterns can vary across different cell lines. This variability accounts for why not all HIF-1α target genes were affected. Co-IP assays indicated no direct interaction between ONECUT3 and HIF-1α. Subsequently, we identified five genes (*NAA10*, *HNF4A*, *TCEB2*, *HDAC6*, and *ELAVL1*) that interact directly with HIF-1α and are regulated by ONECUT3. While HIF-1α has been extensively studied as a classic regulatory factor in signaling pathways, there has been limited progress in clinical research targeting HIF-1α, particularly in CRC. The discovery of ONECUT3 and its regulatory effect on HIF-1α presents significant research potential for developing new drugs targeting HIF-1α.

HDAC6, a class IIB histone deacetylase member, primarily targets α-microtubulin, influencing the cytoskeleton and cell mobility [46]. Its overexpression is associated with tumorigenesis and can act as a prognostic marker [34]. Given its significant role in tumors, HDAC6 has become a potential target for developing antitumor drugs [47]. Inhibition of HDAC6 has been shown to reduce HIF-1-mediated transcription under hypoxic conditions selectively. HDAC6 features two catalytic domains, CD1 and CD2, which are effective lysine deacetylases [46]. The deacetylase activity of HDAC6 contributes to the stabilization and transcriptional activity of HIF-1α [48]. Consequently, we identified HDAC6 as a potential downstream factor of ONECUT3. Molecular biology experiments revealed that knocking down *ONECUT3* significantly inhibited HDAC6 expression and that ONECUT3 directly regulates the transcription of HDAC6. The knockdown of either ONECUT3 or HDAC6 in colon cancer cells led to a marked increase in the acetylation level of HIF-1α. Conversely, overexpressing *ONECUT3* significantly decreased the acetylation level of HIF-1α, thereby enhancing its transcriptional activity. Inhibition of HDAC6 diminished the effects of *ONECUT3* overexpression on HIF-1α acetylation and transcriptional activity. Due to the elevated expression of HDAC family members in various malignant tumors and their diverse carcinogenic mechanisms, the U.S. Food and Drug Administration (FDA) has approved HDAC inhibitors (HDACIs) for treating T-cell lymphoma and multiple myeloma. Currently, various HDACIs are being tested in combination with other antitumor drugs in clinical trials for CRC, showing promising results for combined therapies [49]. For example, at the 2023 European Society for Medical Oncology (ESMO) conference, the Cancer Prevention and Treatment Center of Sun Yat-sen University presented a novel treatment approach for CRC using Sintilimab in conjunction with Bevacizumab and Chidamide, exploring the application of HDACIs alongside anti-vascular therapy and immunotherapy in CRC [50]. Our study indicates that HDAC6 is involved in regulating the Warburg effect in CRC through ONECUT3, providing new evidence for the potential use of HDAC inhibitors in the treatment of CRC.

This study does have some limitations: (1) The investigation into the glycolysis mechanisms is not sufficiently thorough, particularly regarding the molecular mechanisms. In pancreatic cancer, ONECUT3 can promote the process of glycolysis by regulating pyruvate dehydrogenase kinase (PDK) [44]. Furthermore, in myelodysplastic syndromes, overexpression of ONECUT3 causes chromosomal passenger complex (CPC) dysregulation and mitotic defects that affect cell mitosis [51]. Cell cycle-related proteins such as CDK1 and CDC20 can regulate the stability of HIF-1α, and it remains to be demonstrated whether ONECUT3 can regulate HIF-1α by affecting the cell cycle [52]. Therefore, the promotive effect of ONECUT3 on glycolysis requires more in-depth and comprehensive mechanistic studies; (2) The small clinical sample size limits its ability to represent the broader clinical population.

## Conclusions

The upregulation of ONECUT3 in CRC facilitates tumor growth by enhancing the transcriptional activity of HIF-1α through HDAC6-mediated deacetylation. This process activates the transcription of glycolytic genes and enhances the Warburg effect in CRC. Targeting ONECUT3 and its associated signaling pathways may offer promising therapeutic avenues for CRC treatment.

## Methods

### Bioinformatics analysis

COAD mRNA data were downloaded from the official website of TCGA. Unsupervised clustering was performed on the TCGA-COAD data to select a classification that includes glycolysis-related genes for hierarchical clustering [53]. ConsensusClusterPlus was employed for hierarchical clustering was used to classify colon cancer samples into low-glycolytic and high-glycolytic groups. The limma package of R was used to screen for differentially expressed genes in the two groups. Log_2_ |Fold change | ≥ 1 and a significance level of *p* < 0.05 were used as the cut-off value to identify up-regulated genes in the high-glycolysis group. The up-regulated genes were compared with the transcription factor subset (MsigDB: TF) of the Molecular Characterization Database for further analysis. The JASPAR database (http://jaspardev.genereg.net/), ALGGEN database (http://alggen.lsi.upc.es/home.html), and hTFtarget database (http://bioinfo.life.hust.edu.cn/hTFtarget) were used to predict the potential target genes regulated by ONECUT3.

### Cell culture and reagents

We obtained human colon cancer cell lines CACO2, LOVO, SW480, and SW620 from the Institute of Biochemistry and Cell Biology, Chinese Academy of Sciences (Shanghai, China), COLO205, HT29, and the colon immortalized cell line NCM460 from ATCC (American type culture collection). All the cell lines were authenticated (STR profiling) and tested for mycoplasma contamination. The cells were cultured in a suggested standard medium (Gibco, USA) supplemented with 10% fetal bovine serum (FBS, Gibco, USA), 500 units/mL of penicillin, and 200 μg/mL of streptomycin. The cells were maintained at 37 °C with 5% CO_2_. To create a hypoxic environment, the cells were cultured in a hypoxia incubator with an atmosphere containing 1% O_2_, 94% N_2_, and 5% CO_2_ [54]. The reagents used in this study included 2-DG (Sigma-Aldrich, D8375).

### Clinical samples and IHC staining

We utilized paraffin-embedded tissue microarrays, which consisted of cancer tissues from 141 CRC patients who underwent surgeries at Ren Ji Hospital, School of Medicine, Shanghai Jiao Tong University, for immunohistochemical (IHC) analysis of many markers. Additional paratumoral normal tissue and paired tumor histopathology sections from 10 colon cancer patients were used to analyze ONECUT3 expression. All patients included in this study were pathologically diagnosed with CRC and had complete clinicopathological characteristics. None of these patients received any preoperative antitumor therapy, such as chemotherapy or radiotherapy. The specimens were collected with informed consent from all patients and approved by the ethics committee of Renji Hospital (KY2021-120-B). IHC staining was conducted as previously described [28]. The primary antibodies used for IHC staining included ONECUT3 (1:200, abcam, ab181450), HDAC6 (1:200, Immunoway, YT2118), ALDOA (1:100, proteintech, 11217-1-AP), ENO1 (1:2000, proteintech, 11204-1-AP), TPI1 (1:100, proteintech, 10713-1-AP), GLUT1 (1:200, CST, D3J3A/12939S), and HIF-1α (1:100, proteintech, 20960-1-AP). Two investigators blinded to the clinical information assessed the scoring based on the staining intensity.

### Patient survival analysis and database analysis

We extracted the expression data for the ONECUT3 gene and prognostic information from each sample in the TCGA-COAD dataset. Samples with follow-up times shorter than 30 days were excluded. We then performed a log2 (x + 0.001) transformation on each expression value. Subsequently, we obtained data on overall survival (OS), progression-free interval (PFI), disease-specific survival (DSS), and disease-free interval (DFI). To determine the optimal cutoff value for ONECUT3, we utilized the R package maxstat (Maximally selected rank statistics with several *p*-value approximations, version: 0.7–25). Based on this cutoff, patients were categorized into high and low-expression groups. We analyzed the prognostic differences between these two groups using the survfit function from the R package survival. We assessed the significance of these differences using the log-rank test method.

### Quantitative real-time polymerase chain reaction (qRT-PCR)

TRIzol total RNA isolation reagent (share-bio, Shanghai, China) was used to extract total RNA from the indicated cells. The quality and quantity of RNA were determined using a Nanodrop™ spectrophotometer (NanoDrop products, Wilmington, CA). Then, 1 μg of total RNA was reverse transcribed into complementary DNA (cDNA) using an All-in-One First-Strand Synthesis Master Mix (with dsDNase) (share-bio, Shanghai, China). Subsequently, the cDNA product was amplified by PCR on the ViiA 7 Real-Time PCR System (Thermo Scientific) to analyze mRNA expression. The 2*Universal SYBR Green qPCR Premix (share-bio, Shanghai, China) and specific primers were used for qPCR. 18sRNA was used as an internal control. The PCR primer sequences used in this study are listed in Supplementary Table 1. Relative quantification was performed using the comparative 2^–ΔΔCt^ method.

### Western blot analysis and co-immunoprecipitation

Whole-cell protein lysates were obtained using IP lysis buffer (share-bio, Shanghai, China) supplemented with a protease and phosphatase inhibitor (share-bio, Shanghai, China). The cell lysates were separated using 10% sodium dodecyl sulfate-polyacrylamide gel electrophoresis (SDS-PAGE) and subsequently electrophoretically transferred onto PVDF membranes. The membranes were blocked with 5% defatted milk for 1 hour at room temperature (RT), hybridized with primary antibodies overnight at 4 °C, and then incubated with HRP-conjugated secondary antibodies at RT for 60 min. β-actin antibody was used as a loading control. Immunoblots were developed using the Basic Luminol Chemiluminescent Kit (share-bio, Shanghai, China) and the ChemiDoc Touch image system (Bio-Rad). The antibodies used were listed as follows: ONECUT3 (1:500, Abcam, ab181450), HIF1α (1:1000, Abcam, ab2185), GLUT1 (1:1000, Proteintech, 21829-1-AP), ALDOA (1:10000, Proteintech,11217-1-AP), HDAC6 (1: 10000, Abcam, ab133493), and β-actin (1: 5000, Abcam, ab6276). For co-immunoprecipitation, protein lysates obtained as described above were incubated with Pierce Anti-HA Magnetic Beads (Thermo Fisher Scientific, USA, #88836) or Pierce Protein-A/G Magnetic Beads (Thermo Fisher Scientific, USA, #88803), which were already incubated with anti-HIF1α (2 µg, Abcam, ab308433) or anti-IgG (as a negative control, 2 µg, Abcam, ab200699) for 15 min at RT, with rotation for 30 min at RT. The immuno-complexes were washed thrice with TBS-T or PBS-T and then resuspended in 1× SDS-PAGE sample buffer for western blotting analysis.

### Lentivirus production and transfection

To achieve overexpression, plasmids expressing HA-tagged ONECUT3 were constructed by Shanghai Generay Biotech Co., Ltd. The cDNAs encoding full-length human ONECUT3 (NM_001080488.2) with HA tag were synthesized and inserted into the pCDH-CMV-MCS-EF1-Puro vector (Generay, Shanghai, China). For knockdown, lentiviral siRNA negative control and siRNA oligonucleotides targeting human ONECUT3 were designed and synthesized by Genepharma (Shanghai, China). The sequences for the siRNA were as follows: siONECUT3-1: 5’-CGCTGATCGCCATCTTCAAGGAGAA-3’; HDAC6 siRNA sequence: 5’-GGACAACATGGAGGAGGACAATGTA-3’. 293 T packaging cells were used to produce lentivirus, which was subsequently transfected into target cell lines with 6 µg/ml polybrene for 24 h. Transfected cells used for overexpression or knockdown, as well as their control cells, were selected with 5 µg/ml puromycin for 2 weeks. The overexpression or knockdown efficiency of ONECUT3 was assessed using qRT-PCR and western blotting.

### siRNA targeting ONECUT3 or HDAC6

ONECUT3 or HDAC6 specific siRNA and non-targeting control (NTC) siRNA were purchased from Genepharma (Shanghai, China). According to the instructions of jetPRIME® in vitro DNA & siRNA transfection reagent (Polyplus, Shanghai, China), dilute ONECUT3 or HDAC6 specific siRNA (25 nM) or NTC siRNA (25 nM) into 200 µL of jetPRIME® buffer, then add 4 µL jetPRIME® reagent, incubate for 10 to 15 min at RT, add the transfection mix to the cells in serum-containing medium dropwise, and incubate the plate at 37 °C. As mentioned earlier, transfection efficiency was determined by quantitative PCR (qPCR) and immunoblot analysis. Relevant experiments were conducted between 24 and 36 hours after siRNA introduction.

### Colony formation assay

Different types of cells (1 × 10^3^ cells per plate) were seeded in 6-well plates and incubated for approximately 14 days. After the experiments, the formed colonies were washed twice with PBS, fixed with 4% paraformaldehyde for 15 min, and stained with 0.2% crystal violet for 30 min. Colonies larger than 100 μm in diameter were counted for each plate.

### Extracellular acidification rate (ECAR)

The Seahorse XF96 Flux Analyzer (Seahorse Bioscience, Billerica, Massachusetts, USA) was used to measure the real-time extracellular acidification rate (ECAR) of CRC cells in vitro, following the manufacturer’s instructions. Briefly, CRC cells were seeded at a density of 2–3 × 10^4^ cells per well in an XF96-well plate and allowed to attach overnight. Cells were incubated in non-buffered media under basal conditions for 1 hour. Subsequently, they were sequentially injected with 10 mM glucose, 1 mM mitochondrial poison (oligomycin, Sigma-Aldrich, Saint Louis, Missouri, USA), and 80 mM glycolysis inhibitor (2-deoxyglucose, 2-DG, Sigma-Aldrich). ECAR measurement was normalized by total protein content, as demonstrated by the BCA assay. The experimental data was processed using the Seahorse XF96 Wave software.

### Subcutaneous xenograft model

In the colon cancer cell line HT29, *ONECUT3* was knocked down. Approximately 2 × 10^6^ cells from both groups (Sh-*OC3*#1 and Sh-control) were resuspended in 100 μl of PBS and subcutaneously injected into the backs of male Balb/c nude mice (5–6 weeks old). Ten mice were randomly assigned to two groups, with five in each group. After 5 weeks of subcutaneous inoculation, the experiment was concluded, and the mice were euthanized. The tumors were subsequently collected, weighed, and fixed in formalin. All measurements were in a blinded manner. All animal studies received approval from the Animal Care and Use Committee of Shanghai East Hospital, Tongji University School of Medicine. The mice were cared for humanely by the guidelines outlined in the Guide for the Care and Use of Laboratory Animals, prepared by the National Academy of Sciences and published by the National Institutes of Health.

### HIF-1α activity measurement

We quantified HIF-1α activity by using the HIF-1 alpha Transcription Factor Assay Kit (Abcam, ab133104) following the manufacturer’s instructions after extracting the nuclear fraction. Briefly, processed colon cancer cells were collected, and the nuclear and cytoplasmic proteins were extracted by Nuclear and Cytoplasmic Protein Extraction Kit (Beyotime, P0027). The samples were added to the HIF-1α transcription factor plate wells and then incubated overnight at 4 °C. Diluted HIF-1α primary antibody was added to each well and incubated at room temperature for 1 hour. Subsequently, diluted goat anti-rabbit HRP conjugate was added to each well and incubated at room temperature for 1 hour. After adding the stop solution, the HIF-α DNA binding activity level was measured at 450 nm using a microplate reader.

### Chromatin immunoprecipitation (ChIP) assays

ChIP assays were carried out using the EZ-Magna ChIP Assay Kit according to the manufacturer’s protocols (Millipore, 17-10086). Briefly, cells were cross-linked with 1% PFA/PBS at room temperature for 10 minutes. Then, unreacted PFA was eliminated using a 10-fold concentration of glycine. Subsequently, samples were sonicated in lysis buffer to obtain DNA fragments ranging from 200 to 1000 bp. Immunoprecipitation was performed using 5 μg of ONECUT3 or IgG antibodies. Primers targeting the promoter region of the HDAC6 gene were used for quantitative RT-PCR. The results were presented as the relative mRNA expression, calculated by comparing the delta CT values of ONECUT3-specific antibodies with IgG antibodies.

### Luciferase reporter assay

To evaluate the activity of the HDAC6 gene promoter, processed cells and control cells were seeded into 96-well plates and co-transfected with a PTRF luciferase reporter plasmid. The plasmid contained a tandem repeat of the PTRF transcriptional response element, while the Renilla control reporter served as an internal control. After 48 hours, the cells were lysed, and the enzymatic activity of luciferase and Renilla was measured using the Dual-Luciferase Assay kit (Promega, E1910) following the manufacturer’s protocol.

### Statistical analysis

We conducted statistical analyses using SPSS 19.0 for Windows and GraphPad Prism 7 software (San Diego, CA). The results were reported as mean ± standard deviation (SD) and compared using a two-tailed, unpaired Student’s t-test or one-way ANOVA. *P*-value of < 0.05 was considered statistically significant. *P < 0.05, **P < 0.01, ***P < 0.001, ns, not significant.

## Supplementary information

Supplementary materials

A reproducibility checklist

Original data of western blotting.

## Data Availability

Data is available within the article.
