# Supplementary material for: Transcription factor ONECUT3 regulates HDAC6/HIF-1α activity to promote the Warburg effect and tumor growth in colorectal cancer
Source: Cell Death Dis. 2025 Mar 3;16(1):149. doi: 10.1038/s41419-025-07457-8 (PMC11876336; doi:10.1038/s41419-025-07457-8)
Supplement: Supplementary file 1 — Supplementary materials [file 41419_2025_7457_MOESM1_ESM.docx]

Supplementary materials


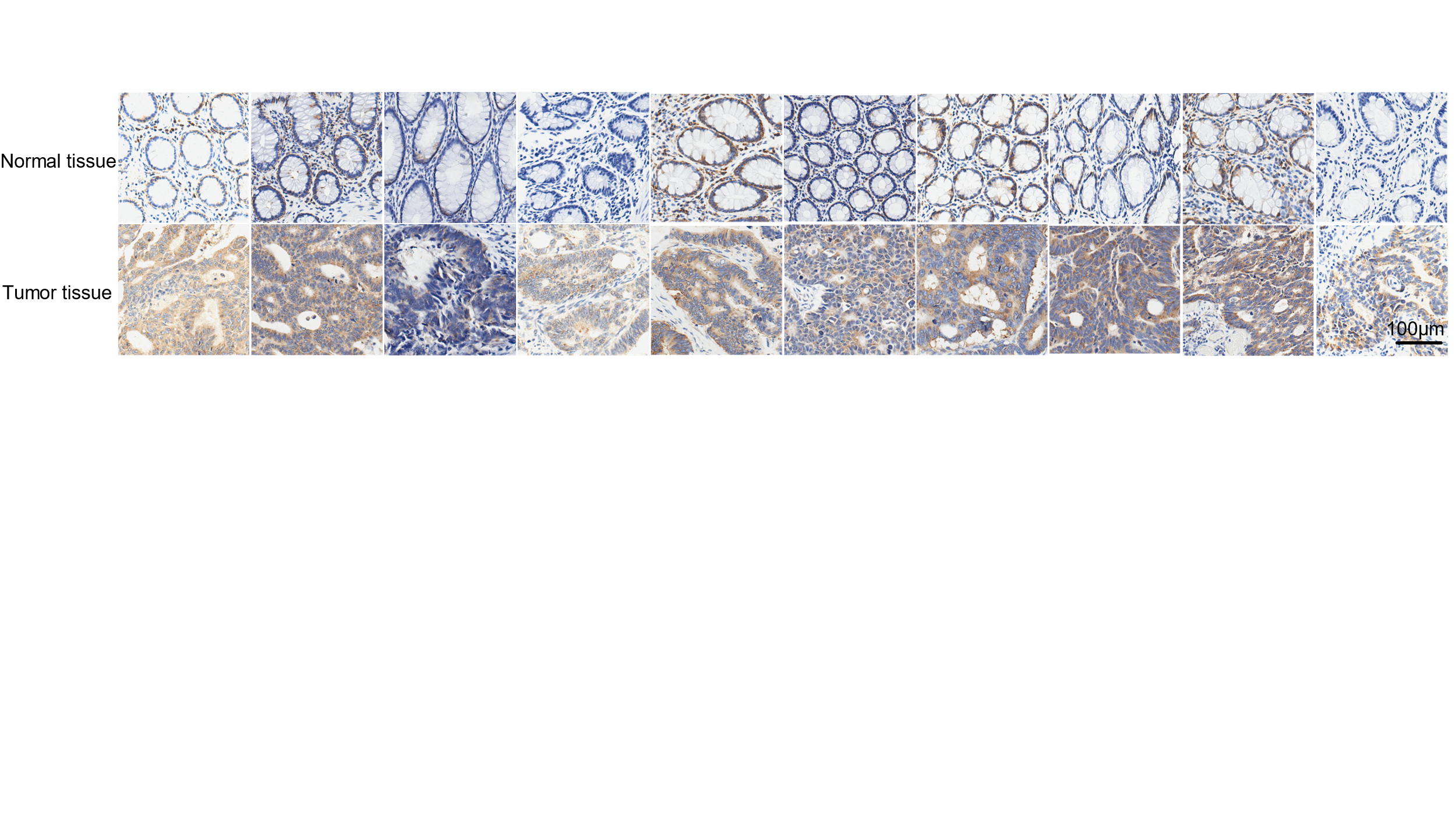


Supplementary Figure 1: Immunohistochemical staining of ONECUT3 in paired samples from 10 colon cancer patients. The top row shows relatively normal colon tissue away from the tumor, and the bottom row shows the corresponding tumor tissue from the same patient.


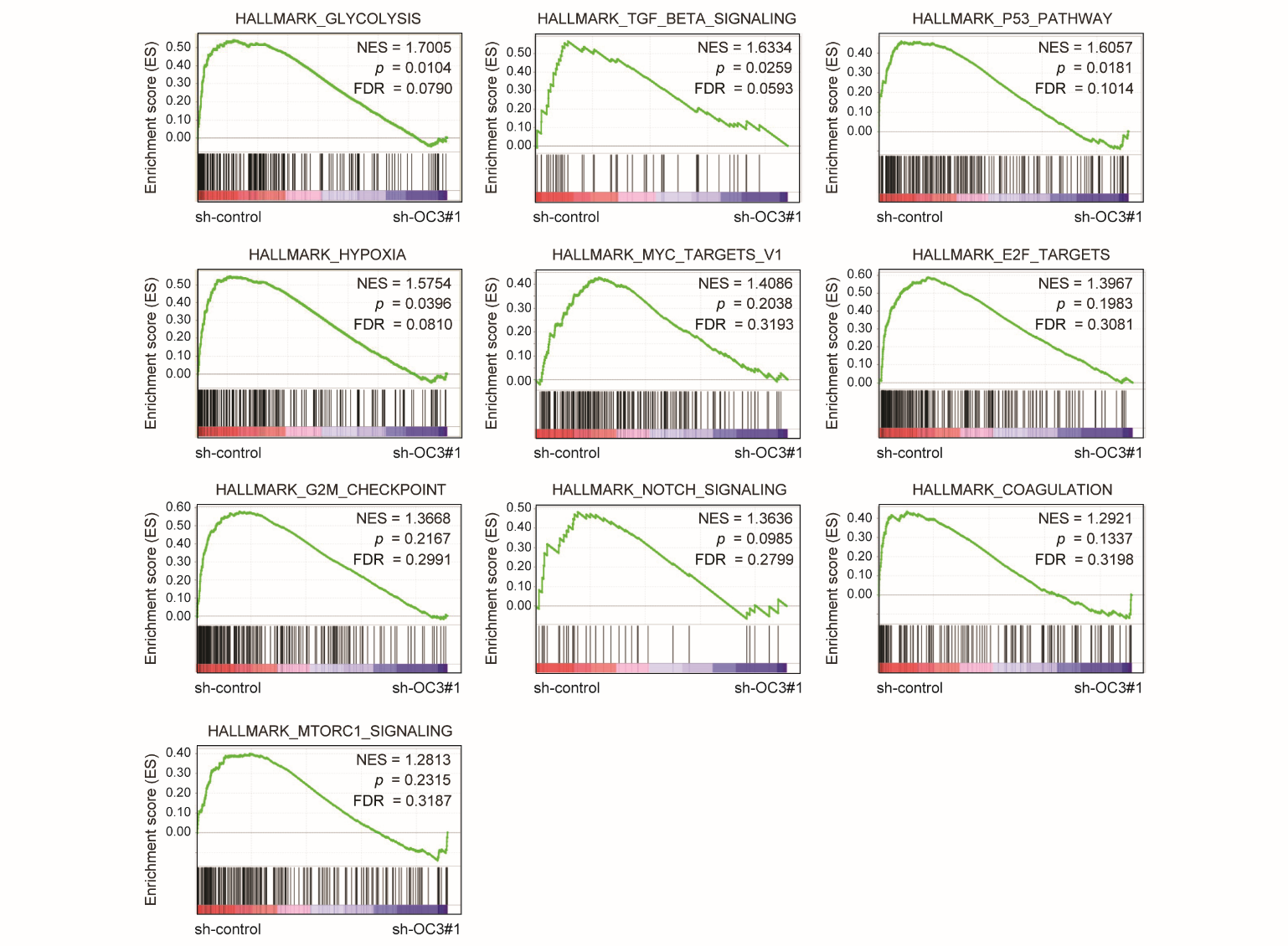


Supplementary Figure 2: The top 10 GSEA-enriched pathways related to ONECUT3.

Supplementary Table 1

| Gene name | Forward primer(5’-3’) | Reverse primer(3’-5’) |
| --- | --- | --- |
| *ONECUT3* | ACCCCCACAGAAGGCTCTAT | TGTGCTGGAGGTGTTGATCC |
| *SLC2A1* | TCTGGCATCAACGCTGTCTTC | CGATACCGGAGCCAATGGT |
| *HK2* | TTGACCAGGAGATTGACATGGG | CAACCGCATCAGGACCTCA |
| *GPI* | GGAGACCATCACGAATGCAGA | TAGACAGGGCAACAAAGTGCT |
| *PFKL* | GCTGGGCGGCACTATCATT | TCAGGTGCGAGTAGGTCCG |
| *ALDOA* | GCTGTCACTGGGATCACCTTC | GCTCGGAGTGTACTTTCCTTGA |
| *GAPDH* | GGAGCGAGATCCCTCCAAAAT | GGCTGTTGTCATACTTCTCATGG |
| *PGK1* | TGGACGTTAAAGGGAAGCGG | GCTCATAAGGACTACCGACTTGG |
| *PGAM1* | GTGCAGAAGAGAGCGATCCG | CGGTTAGACCCCCATAGTGC |
| *ENO1* | TGGTGTCTATCGAAGATCCCTT | CCTTGGCGATCCTCTTTGG |
| *ENO2* | AGCCTCTACGGGCATCTATGA | TTCTCAGTCCCATCCAACTCC |
| *LDHA* | ATGGCAACTCTAAAGGATCAGC | CCAACCCCAACAACTGTAATCT |
| *PKM2* | ATGTCGAAGCCCCATAGTGAA | TGGGTGGTGAATCAATGTCCA |
| *HDAC6* | AAGAAGACCTAATCGTGGGACT | GCTGTGAACCAACATCAGCTC |
| *HIF1A* | GAACGTCGAAAAGAAAAGTCTCG | CCTTATCAAGATGCGAACTCACA |
