# Supplementary figures and images for: Transcription factor ONECUT3 regulates HDAC6/HIF-1α activity to promote the Warburg effect and tumor growth in colorectal cancer
Source: Cell Death Dis. 2025 Mar 3;16(1):149. doi: 10.1038/s41419-025-07457-8 (PMC11876336; doi:10.1038/s41419-025-07457-8)

Figure 2B

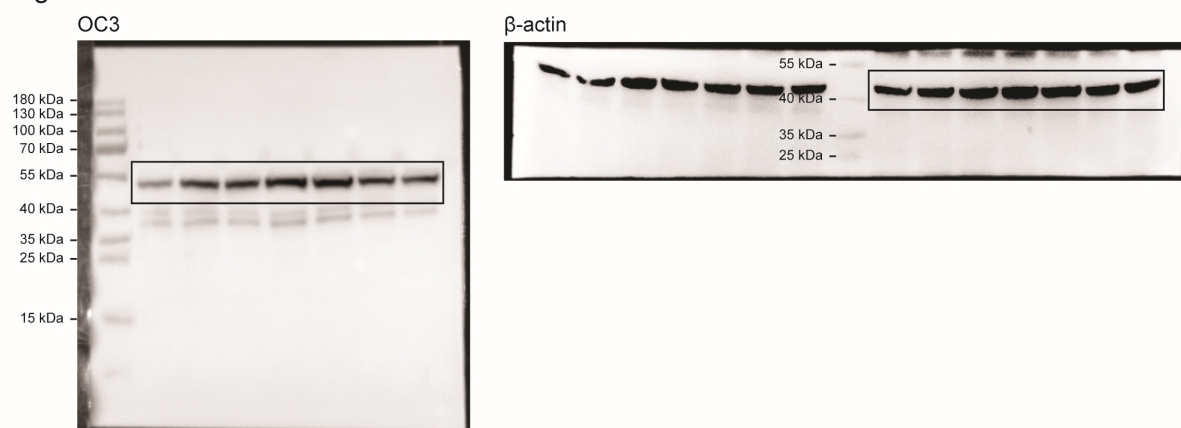

Figure 2C

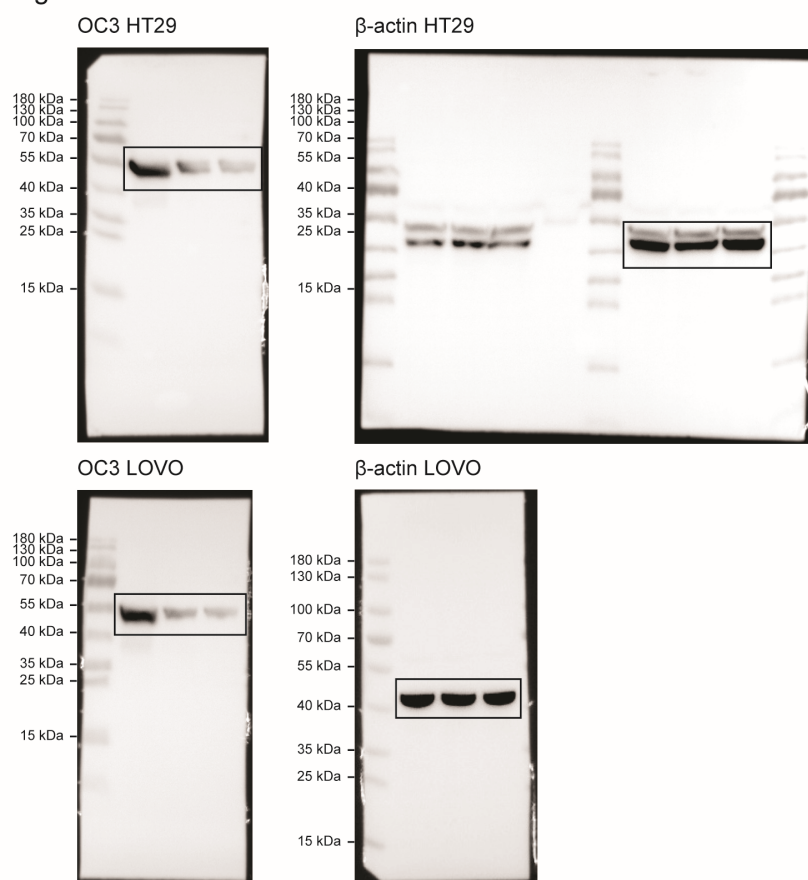

Figure 2E

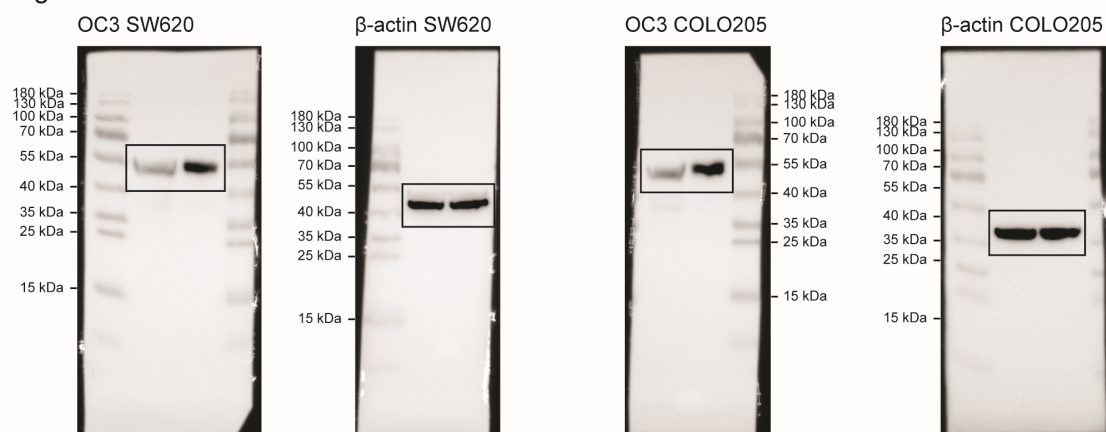

Figure 4C

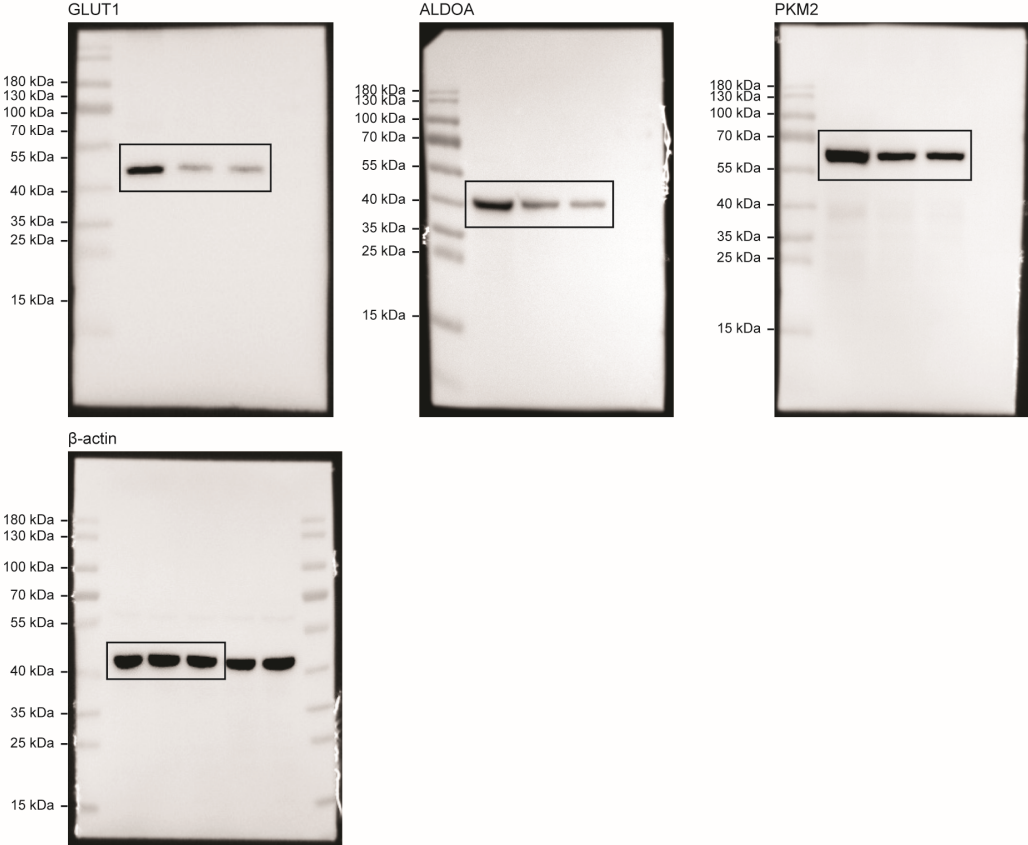

Figure 4E

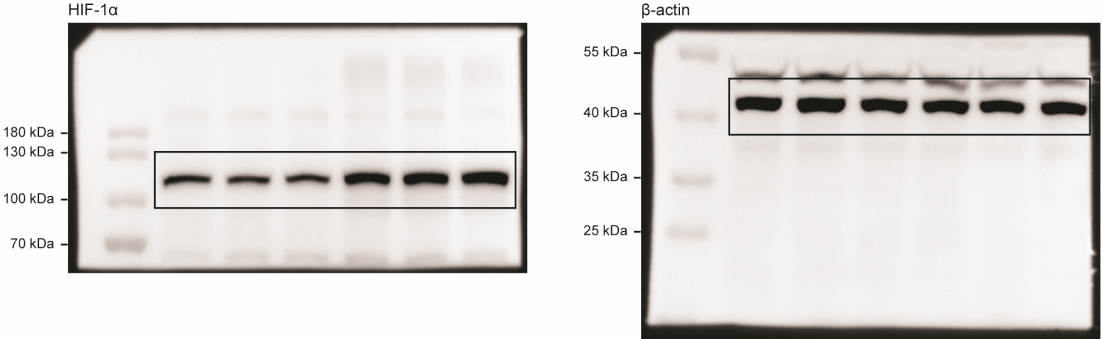

Figure 4G

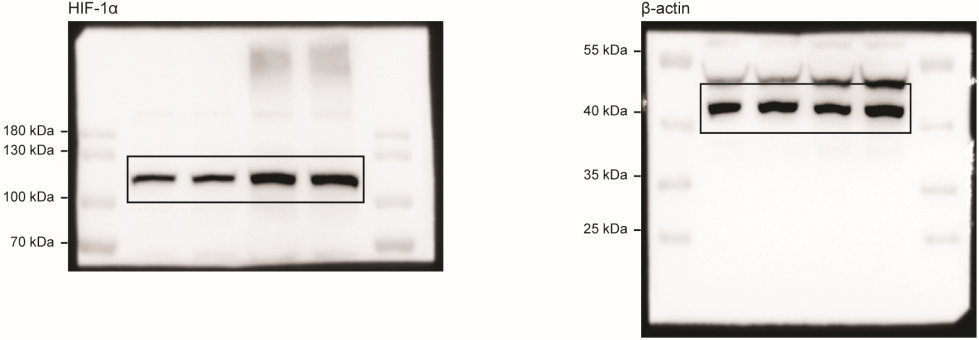

Figure 5D

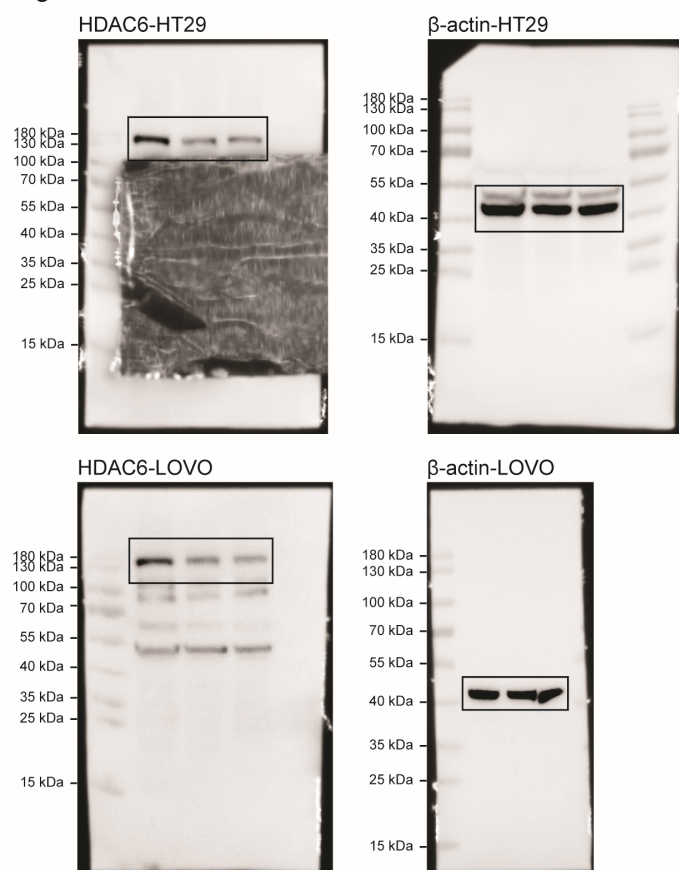

Figure 6A

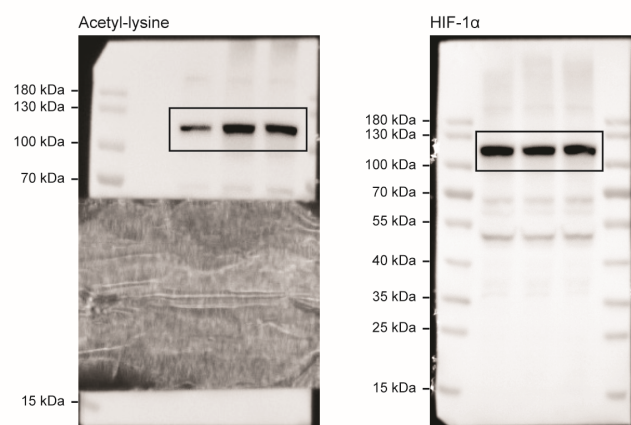

Figure 6B

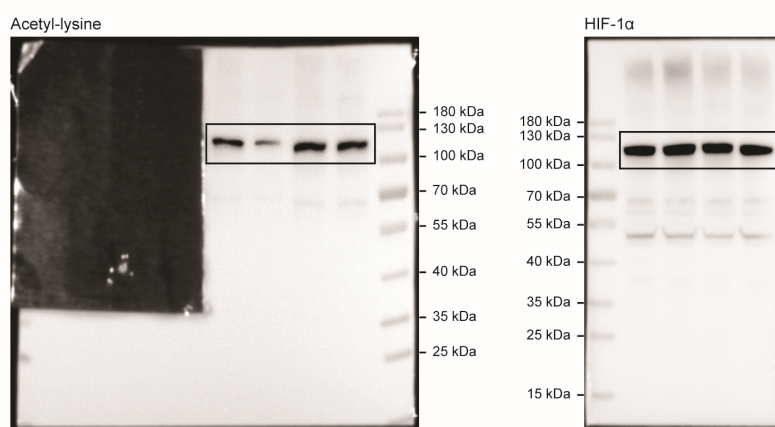

Supplement: Supplementary file 3 — Original data of western blotting. [file 41419_2025_7457_MOESM3_ESM.pdf]
